# Enhancing Comparative Effectiveness Research With Automated Pediatric Pneumonia Detection in a Multi-Institutional Clinical Repository: A PHIS+ Pilot Study

**DOI:** 10.2196/jmir.6887

**Published:** 2017-05-15

**Authors:** Stephane Meystre, Ramkiran Gouripeddi, Joel Tieder, Jeffrey Simmons, Rajendu Srivastava, Samir Shah

**Affiliations:** ^1^ Medical University of South Carolina Charleston, SC United States; ^2^ Department of Biomedical Informatics University of Utah Salt Lake City, UT United States; ^3^ Seattle Children’s Hospital and University of Washington Seattle, WA United States; ^4^ Cincinnati Children’s Hospital Medical Center Cincinnati, OH United States; ^5^ Department of Pediatrics University of Utah Salt Lake City, UT United States; ^6^ Primary Children's Hospital Salt Lake City, UT United States

**Keywords:** natural language processing, pneumonia, bacterial, medical informatics, comparative effectiveness research

## Abstract

**Background:**

Community-acquired pneumonia is a leading cause of pediatric morbidity. Administrative data are often used to conduct comparative effectiveness research (CER) with sufficient sample sizes to enhance detection of important outcomes. However, such studies are prone to misclassification errors because of the variable accuracy of discharge diagnosis codes.

**Objective:**

The aim of this study was to develop an automated, scalable, and accurate method to determine the presence or absence of pneumonia in children using chest imaging reports.

**Methods:**

The multi-institutional PHIS+ clinical repository was developed to support pediatric CER by expanding an administrative database of children’s hospitals with detailed clinical data. To develop a scalable approach to find patients with bacterial pneumonia more accurately, we developed a Natural Language Processing (NLP) application to extract relevant information from chest diagnostic imaging reports. Domain experts established a reference standard by manually annotating 282 reports to train and then test the NLP application. Findings of pleural effusion, pulmonary infiltrate, and pneumonia were automatically extracted from the reports and then used to automatically classify whether a report was consistent with bacterial pneumonia.

**Results:**

Compared with the annotated diagnostic imaging reports reference standard, the most accurate implementation of machine learning algorithms in our NLP application allowed extracting relevant findings with a sensitivity of .939 and a positive predictive value of .925. It allowed classifying reports with a sensitivity of .71, a positive predictive value of .86, and a specificity of .962. When compared with each of the domain experts manually annotating these reports, the NLP application allowed for significantly higher sensitivity (.71 vs .527) and similar positive predictive value and specificity *.*

**Conclusions:**

NLP-based pneumonia information extraction of pediatric diagnostic imaging reports performed better than domain experts in this pilot study. NLP is an efficient method to extract information from a large collection of imaging reports to facilitate CER.

## Introduction

Community-acquired pneumonia (CAP) is a leading cause of hospitalization among children in the United States [1,2]. Despite this prevalence, the effectiveness of common management strategies [3] is unknown. Multicenter studies using administrative data are inexpensive to conduct and could help compare treatment effectiveness and overcome the challenge of measuring adverse outcomes [4,5]. However, these studies are limited by the potential for subject misclassification. International Classification of Diseases, 9th revision, Clinical Modification (ICD-9-CM) discharge diagnosis codes are commonly used to identify patients [4,5]. Improper use of these codes may lead to false positive or false negative cases [6]. In studies of pediatric CAP, this might lead to systematic biasing by inadvertently including patients without pneumonia or excluding patients with pneumonia in the study cohort [7]. Furthermore, use of these discharge diagnosis codes only precludes more accurate risk adjustment than might be available through admission chest radiograph results, for example [8].

The PHIS+ repository augments the Pediatric Health Information System (PHIS), an administrative database from the Children’s Hospital Association, with clinical data [9]. PHIS+, consists of laboratory [9] and microbiological testing results [10], as well as imaging reports from 6 pediatric hospitals across multiple care settings (inpatient, outpatient, emergency department, and ambulatory surgery) over a 5-year study period. The clinical data in the PHIS+ repository are standardized and harmonized using biomedical terminologies and common data models. But, unlike laboratory results, which are available in discrete formats for comparative effectiveness research analyses, imaging reports are available only in narrative clinical text and lack standardization in structure and format. To allow for efficient and rapid access to these data, we developed a Natural Language Processing (NLP) application to determine the diagnosis of bacterial pneumonia from pediatric diagnostic imaging reports by extracting pneumonia characteristics (ie, presence, symmetry, and size of pleural effusion and pulmonary infiltrate) [11].

NLP has been used to extract different types of clinical information from various sources of narrative text in adult patients [12]. Studies have applied Bayesian networks and NLP to detect bacterial pneumonia in adults [13], and several used an NLP application called MedLEE [14] to extract community-acquired pneumonia severity scores in adults [15] and pneumonia information from chest radiology reports in a neonatal intensive care unit [16], or to identify patients with tuberculosis [17]. Recent efforts applied NLP to extract pneumonia information from radiology reports in an adult intensive care unit [18], detect probable pneumonia cases and help manual chart review [19], and also included electronic health record structured data to detect pneumonia cases [20]. These studies reported accuracy metrics with large variations, sensitivity ranging from .45 to .95, and positive predictive value (PPV) from .075 to .86 (best PPV was .86 with a sensitivity of .75 [18], and best sensitivity was .95 with a PPV of .78 [13]). They typically focused on only one type of clinical note, at only one health care organization or hospital, and included the complete development of large complex NLP systems. Only one of these prior studies included children evaluated for pneumonia [19], but it required a manual review of a subset of the radiology reports already analyzed by the NLP system. A good recent review of NLP applications to radiology reports can be found in [21]. The goal of this study was to develop an automated, scalable, and accurate method to determine the presence or absence of pneumonia in children, using a large variety of chest imaging reports from the newly developed PHIS+ repository in order to facilitate the conduct of adequately powered comparative effectiveness research aimed for treatment options of hospitalized children.

## Methods

### Study Sites

Six free-standing children’s hospitals were included: Boston Children’s Hospital (Boston, MA, USA); Children’s Hospital of Philadelphia (Philadelphia, PA, USA); Children’s Hospital of Pittsburgh (Pittsburgh, PA, USA); Cincinnati Children’s Hospital Medical Center (Cincinnati, OH, USA); Primary Children’s Hospital, Intermountain Healthcare (Salt Lake City, UT, USA); and Seattle Children’s Hospital (Seattle, WA, USA).

### Reference Standard Preparation

The imaging procedures from the six contributing hospitals in the PHIS+ repository were already mapped to Current Procedural Terminology (CPT) codes [22]. We first selected relevant chest diagnostic imaging (chest radiograph, computerized tomography, and ultrasound) procedure CPT codes (see Multimedia Appendix 1), and then extracted a stratified random collection of imaging study reports mapped to these CPT codes. One report was extracted for each randomly selected patient. A preliminary power analysis indicated that a selection of 270 imaging reports would allow a 95% CI of ±4% width with an expected sensitivity of 90%, assuming mention of pneumonia in 25% of the reports (pneumonia is the information we extracted mentioned the least frequently). A total of 282 reports were eventually selected, deidentified using De-ID software (DE-ID Data Corp) [23] and provided as plain text files for NLP-based information extraction.

### Reference Standard Annotation

**Figure 1:**
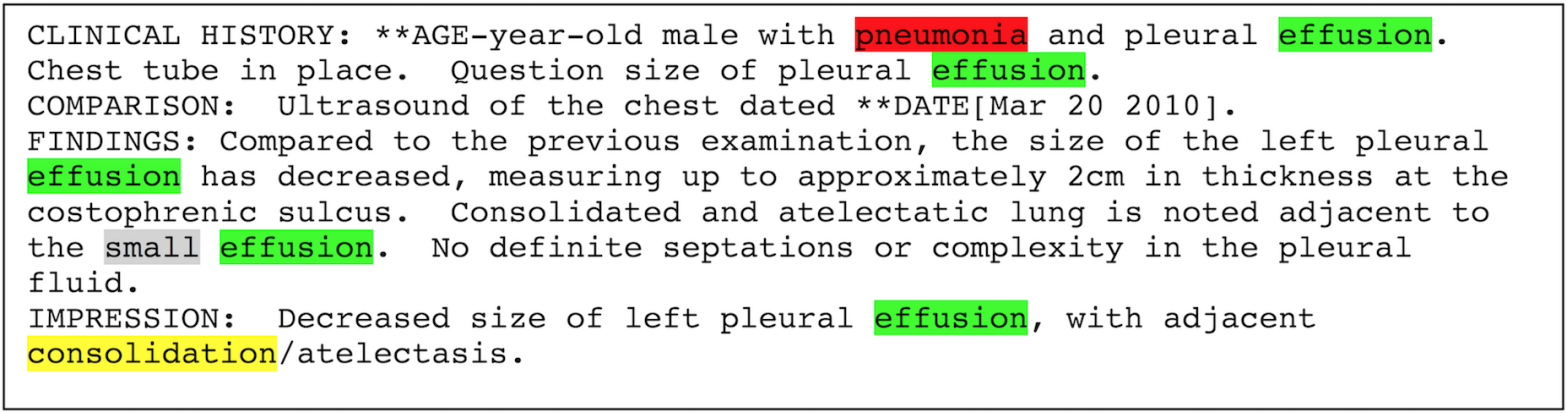
Diagnostic imaging report annotations example.

The 282 deidentified diagnostic imaging reports were annotated by domain experts to evaluate the pneumonia information extraction application. Annotations included all mentions of pulmonary infiltrate, their local context (eg, negation, as in “no infiltrate”), and their symmetry (ie, unilateral or bilateral); pleural effusions, their local context, and their size (ie, small or moderate or large); mentions of pneumonia and their local context (eg, “consistent with pneumonia” or “no evidence of pneumonia”); and whether the report supported the diagnosis of bacterial pneumonia (Figure 1).

**Figure 2:**
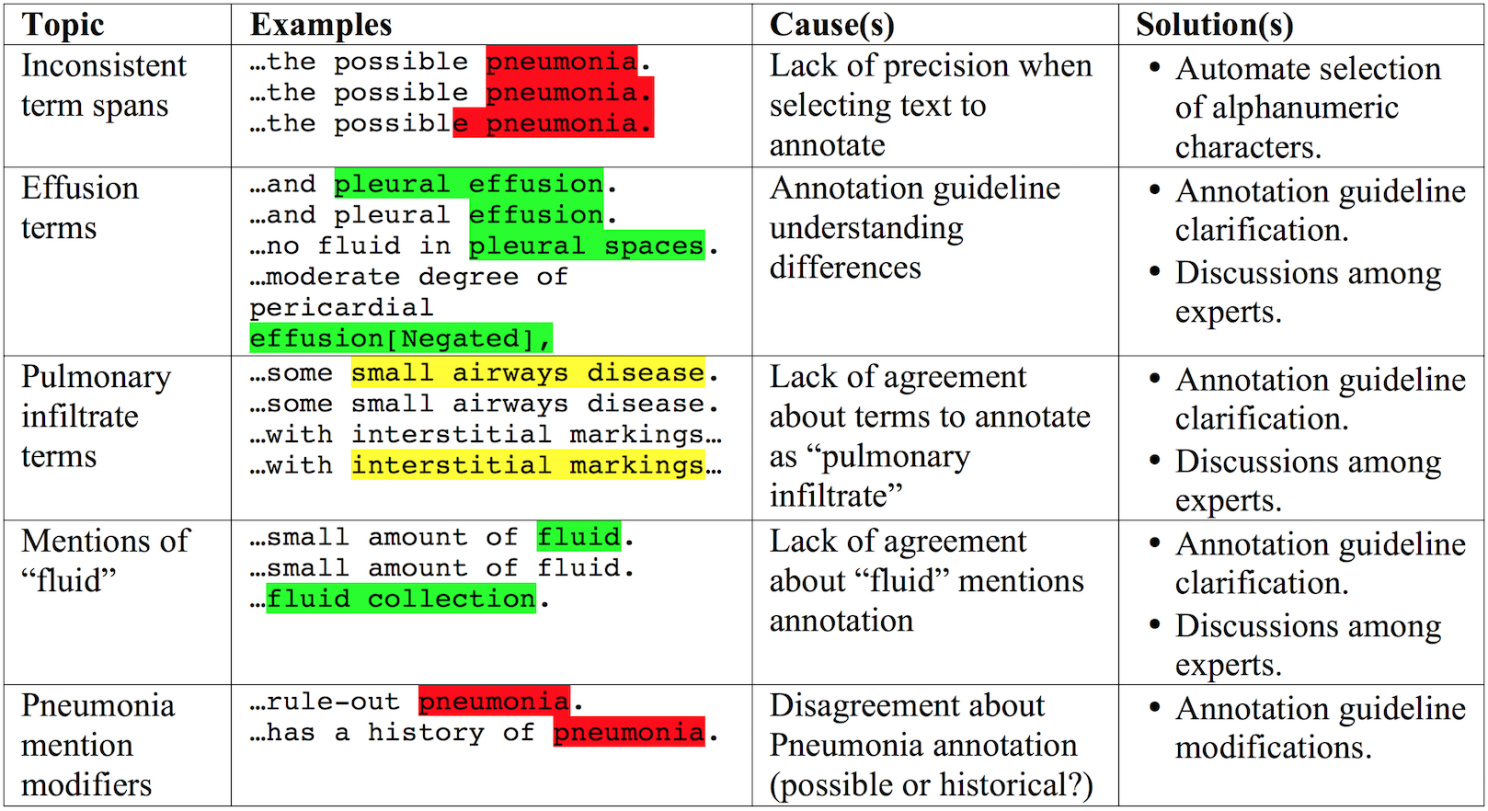
Examples of domain expert annotation disagreements.

The domain experts, three attending pediatric hospital medicine physicians, were trained while also iteratively refining the annotation instructions on the basis of their experience. They first annotated a set of 15 reports, with low interannotator agreement. Examples of disagreements between domain experts are listed in Figure 2.

After having discussed disagreements and updated the annotation instructions, they annotated a second set of 10 other reports and reached fair agreement (pairwise proportions of agreement: .65-.78 for infiltrates, .12-.7 for effusions, and .43-.74 for mentions of pneumonia). Finally, after a final round of disagreement discussions and instructions refinement, they annotated 10 new reports and reached excellent agreement (.96-.98 for infiltrates, .94-1 for effusions, and .92-1 for mentions of pneumonia). The training phase then ended, and annotation of the complete 282 reports collection followed (including reannotation of the initial 15+10+10 reports). At this stage, the rare disagreements were discussed among all domain experts to reach consensus for the reference standard. The annotated information included the following (Figure 1; Final annotation guideline in Multimedia Appendix 2):

- Mentions of “pneumonia” (or synonyms—eg, “pneumonitis”), without adjectives (except if required to define the concept; eg, “lung infection” needs “lung” to be precise enough).
- Mentions of “pleural effusion” (or synonyms—eg, “empyema”; or terms that imply the existence of a pleural effusion if “pleural effusion” or a synonym is not mentioned—eg, “loculation,” “free fluid”), without adjectives.
- Mentions of “pulmonary infiltrate” (or synonyms like “opacity,” “consolidation”), without adjectives or remote synonyms like “small airways disease,” “interstitial markings,” “peribronchial thickening,” or “atelectasis.”
- Context surrounding each pneumonia, effusion, or infiltrate annotation (referred to as “local context”) was annotated as *present* (ie, affirmed, not negated, current), *absent* (ie, negated, excluded), *speculative* (ie, hypothetical, a possibility, to rule it out), or *historical* (ie, in the past, not current anymore).
- Pleural effusion size was annotated as *small*, *moderate-large*, or *not mentioned*.
- Symmetry of infiltrates was annotated as *unilateral*, *bilateral*, or *not mentioned*.
- Overall, each report was annotated as to whether it did or did not generally support the diagnosis of bacterial pneumonia (true or false).

### Clinical Information Extraction Application Development

**Figure 3:**
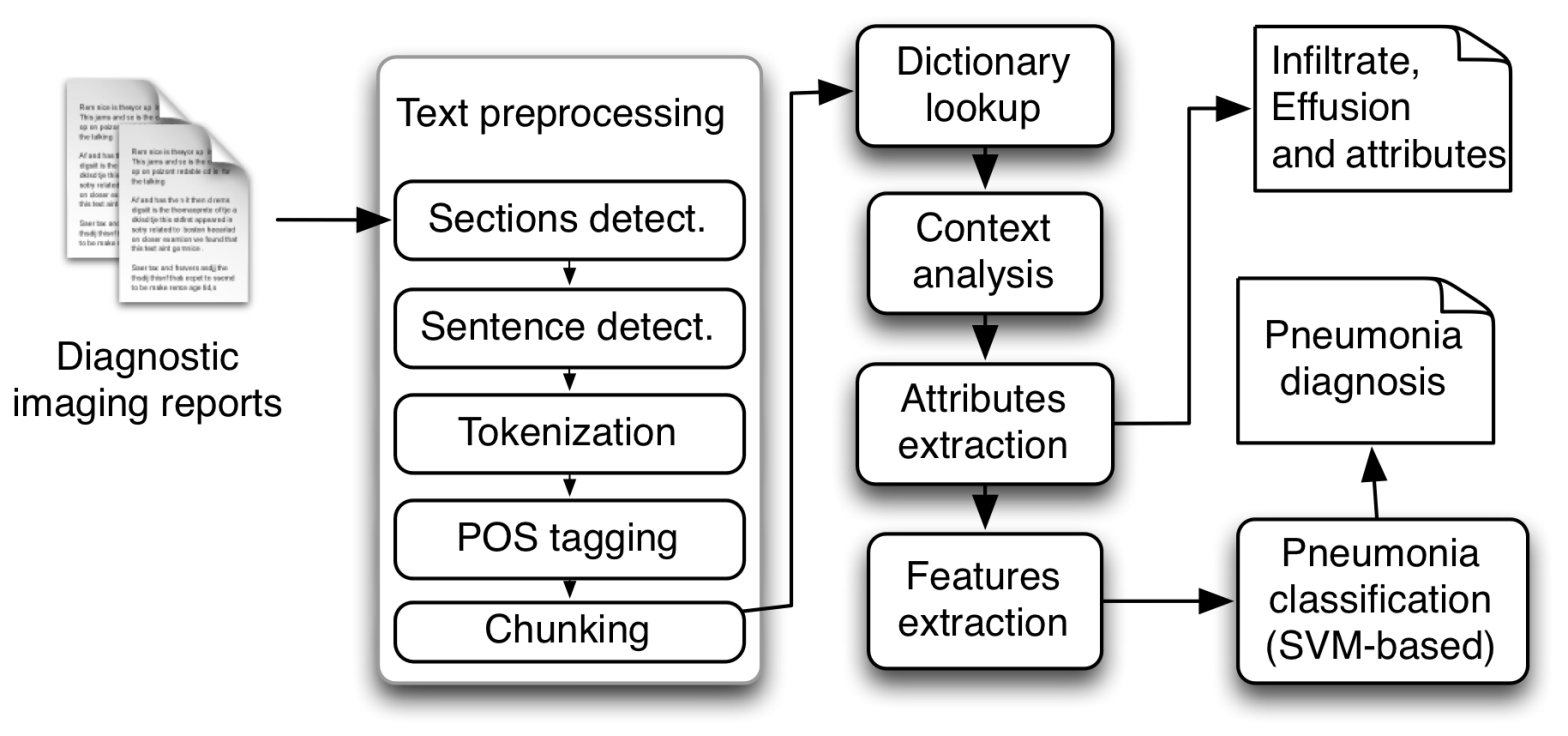
Components of the pneumonia clinical information extraction application.

We developed an application based on NLP to automate the extraction of information. This application was based on the Apache UIMA (Unstructured Information Management Architecture) framework [24] using components either developed specifically for this application or adapted from another NLP application: Textractor [25]. Components included text preprocessing (sections detection, lists annotation, sentence segmentation, tokenization, part-of-speech tagging, and chunking), dictionary look-up, local context analysis, annotation attributes and patient information (hospital and patient code) extraction, machine learning features extraction, and the final classification (Figure 3).

During text preprocessing, sections were detected using a collection of regular expressions representing possible headers for patient history sections. Lists were also detected using regular expressions, and their entries segmented as individual sentences. Segmentation of the text in sentences was adapted from Textractor, which is based on a machine learning algorithm (maximum entropy, MaxEnt [26]). Sentences are then “tokenized,” split in words or other meaningful groups of alphabetical or numeric characters. Each token is then assigned a part-of-speech tag with another module adapted from Textractor that is based on maximum entropy (itself adapted from OpenNLP [26]). Finally, noun phrase “chunks” are detected with a third module adapted from Textractor, which is also based on maximum entropy (also originally adapted from OpenNLP [26]).

The dictionary lookup module searches a list of terms for matches with the noun phrase “chunks” detected in the text. The list of terms (ie, dictionary) was originally based on a subset of the Unified Medical Language System (UMLS) Metathesaurus [27] filtered by semantic type to include only disease or syndrome, finding, or pathologic function. This dictionary was later replaced with a list of terms built manually by clinicians (based on their domain knowledge), an approach that allowed for improved accuracy.

The local context analysis was based on the ConText algorithm [28], as implemented in Textractor. This algorithm looks for keywords that indicate local context such as negation (eg, denied, no, absent), and then assigns this context to concepts found in a window of words following or preceding the keyword. For example, in the sentence “Findings consistent with viral or reactive airways disease without focal pneumonia,” the keyword “without” indicates negation and precedes the annotated concept “pneumonia,” which will therefore be considered negated, or absent.

The extraction of annotation attributes (effusion size and infiltrate symmetry) and patient information (hospital and patient code) was based on a set of regular expressions developed specifically and implemented similarly to ConText, assigning these attributes to the appropriate annotated concepts.

Finally, the classification of reports as supporting the diagnosis of bacterial pneumonia (or not) was based on a Support Vector Machine (SVM) classifier with lexical and semantic features. These features included a “bag-of-words” (ie, list of words occurring more than once in our reports collection, without stopwords like “and,” “from,” “each”) and the annotated concepts with their attributes (eg, “pleural effusion” annotation with “small” quantity attribute). The classifier was an implementation of LIBSVM [29], with the radial basis function (RBF) kernel.

### Application Performance Improvements

When initially evaluating the pneumonia classification accuracy, sensitivity was not satisfactory. Therefore, we compared several different machine learning algorithms, refined parameters for the SVM, and filtered the machine learning features (bag-of-words), as well as the dictionaries used by our application.

Machine learning algorithms compared included decision trees, rule learners, naïve Bayes, Bayesian networks, and SVMs, all implemented in the Weka software (version 3.7; University of Waikato, New Zealand) [30]. Features used were the same with each algorithm and included the annotated concepts, and their attributes and local context. Refining the SVM parameters (ie, the penalty parameter *C*, and the radial basis function parameter gamma; final values allowing for best accuracy: *C*=11.5, gamma=.1) consisted in realizing a grid search for selecting the best values of these parameters (using the Grid Parameter Search tool available with LIBSVM).

The “bag-of-words” is an important set of features for machine learning, and the original version included 2103 different words. Even after excluding stopwords, most remaining words have no meaning associated with the diagnosis or radiological signs of pneumonia. To focus our classification on more meaningful words for our task, we manually reviewed all words in the initial bag-of-words (named BOW0) and created three versions with increasing levels of domain specificity. The first refined bag-of-words (BOW1) included 99 words, the second (more specific) bag-of-words (BOW2) included 37 words, and the third (most specific) bag-of-words (BOW3) included only 23 words. The three refined bag-of-words are listed in Multimedia Appendix 3. All were annotated as unigrams.

Finally, refining our dictionary of terms focused on mentions of pulmonary infiltrate, removing terms that caused many false positive matches, but few correct matches.

### Performance Evaluation Approach

We used a cross validation approach with 5 “folds” for training and validation. This approach starts with random partition of our collection of 282 notes into 5 subsets of approximately the same size. Then, one subset is retained for testing and the remaining four subsets are used for training. This process is repeated 5 times (ie, “folds”), with each subset used only once for testing. In each “fold,” we compared the information extraction application output with the manual reference standard annotations, and classified each annotation as true positive (application output matches the reference standard), false positive (application output not found in the reference standard), or false negatives (reference standard annotation missed by the application). We also counted true negatives for the overall classification when the reference standard and the application both classified the report as not supporting the diagnosis of bacterial pneumonia. Finally, we used counts of true positives, true negatives, false positives, and false negatives, and computed various accuracy metrics at the end of the whole process (not after each fold and then averaged across folds). Accuracy metrics included sensitivity (ie, recall), positive predictive value (ie, precision), the *F*_1_-measure (a harmonic mean of sensitivity and positive predictive value [31]), and the accuracy (proportion of agreement) of the local context category and the attributes category (effusion size and infiltrate symmetry).

For the concept-level evaluation, application automatic annotations and reference standard manual annotations were compared and considered a match when the annotated text overlapped exactly (except preceding or following white space or punctuation) and the annotated information categories (eg, “Effusion”) were the same. For the document-level evaluation, reports were classified as supporting the diagnosis of bacterial pneumonia or not. They were considered a match when their binary classification corresponded to the reference standard classification. For document-level evaluation of domain experts, their initial classification (ie, before adjudication of differences between annotators and reference standard development) were compared with final reference standard classifications.

## Results

### Reference Standard Development

The 282 radiology imaging reports annotated, originated from each of the 6 health care organizations in approximately the same numbers (48 from the Boston Children’s Hospital, 48 from the Children’s Hospital of Philadelphia, 47 from the Children’s Hospital of Pittsburgh, 48 from the Cincinnati Children’s Hospital Medical Center, 47 from the Primary Children’s Hospital, and 44 from the Seattle Children’s Hospital). Annotations included 72 mentions of pneumonia or synonyms (0.255 per report on average), 312 mentions of pulmonary infiltrate or synonyms (1.106), and 369 mentions of pleural effusion or synonyms (1.309). Among the 282 reports, 24.5% (69/282) supported the diagnosis of bacterial pneumonia. Agreement among annotators for the 247 (282 minus 35 reports used for annotators training) not previously seen imaging reports reached 82 of 121 pneumonia mentions (67.8%), 502 of 610 infiltrate mentions (82.3%), and 526 of 670 effusion mentions (78.5%).

### Performance at the Concept Level

Concepts evaluated here included the automatic annotations by our application of mentions of pneumonia, pleural effusion, pulmonary infiltrate, and corresponding local context and attributes. The average sensitivity and positive predictive value were approximately 93-94%, with higher accuracy for mentions of pneumonia, and lower accuracy for mentions of pleural effusion (Table 1). The local context was correct in about 92% (65/71) to 94.1% (272/289) of the cases, and the attribute category in about 72.3% (209/289) to 92.5% (321/347) of the cases.

**Table 1:** Concept level accuracy evaluation results.

Metrics	Terms mentioned in radiology imaging reports			
	Pneumonia	Infiltrate	Effusion	All included terms
True positives	71	289	347	707
False positives	0	20	37	57
False negatives	1	23	22	46
Sensitivity	.986	.926	.940	.939
Positive predictive value	1.000	.935	.904	.925
*F*_1_-measure^a^	.993	.931	.922	.932
Context accuracy	.916	.941	.931	.929
Attribute accuracy	N/A^b^	.723	.925	.824

^a^*F*_1_-measure is a harmonic mean of sensitivity and positive predictive value [31].

^b^N/A: not applicable.

### Performance at the Document Level

This classification was evaluated with various configurations of our application. Sensitivity was quite low (.42) with our initial configuration (Table 2), motivating us to experiment with the aforementioned performance improvement approaches.

When using the SVM classifier with all features (ie, concepts with local context and attributes, and bag-of-words), the more specific bag-of-words (BOW2 and BOW3) allowed for higher positive predictive value and specificity, but sensitivity was the highest at .652 with the least filtered bag-of-words (BOW1). The configuration allowing for the highest sensitivity and *F*_1_-measure was based on the least filtered bag-of-words and a refined dictionary (Best system in Table 2).

We also compared different machine learning algorithms with a limited set of features (ie, no bag-of-words as not all algorithms tested could use it). Most of them allowed for higher sensitivity than the SVM algorithm (as implemented in Weka sequential minimal optimization [SMO] [32]), but their positive predictive value was always lower (see Multimedia Appendix 4).

**Table 2:** Document-level classification results.

Metrics	BOW0^a^	BOW1^b^	BOW2^c^	BOW3^d^	Best system^e^ (95% CI)	Domain experts average
True positives	29	45	31	30	49	36
True negatives	207	200	210	209	205	206
False positives	6	13	3	4	8	7
False negatives	40	24	38	39	20	33
Sensitivity	.420	.652	.449	.435	.710 (.683-.737)	.527
Positive predictive value	.829	.776	.912	.882	.860 (.833-.886)	.848
*F*_1_ measure	.556	.709	.602	.583	.778	.650
Specificity	.972	.939	.986	.981	.962 (.951-.974)	.966
Accuracy	.837	.869	.855	.847	.901 (.883-.918)	.862

^a^ BOW0: Initial bag-of-words.

^b^BOW1: First refined bag-of-words.

^c^BOW2: Second (more specific) refined bag-of-words.

^d^BOW3: Third (most specific) refined bag-of-words.

^e^BOW1 with refined dictionary.

**Figure 4:**
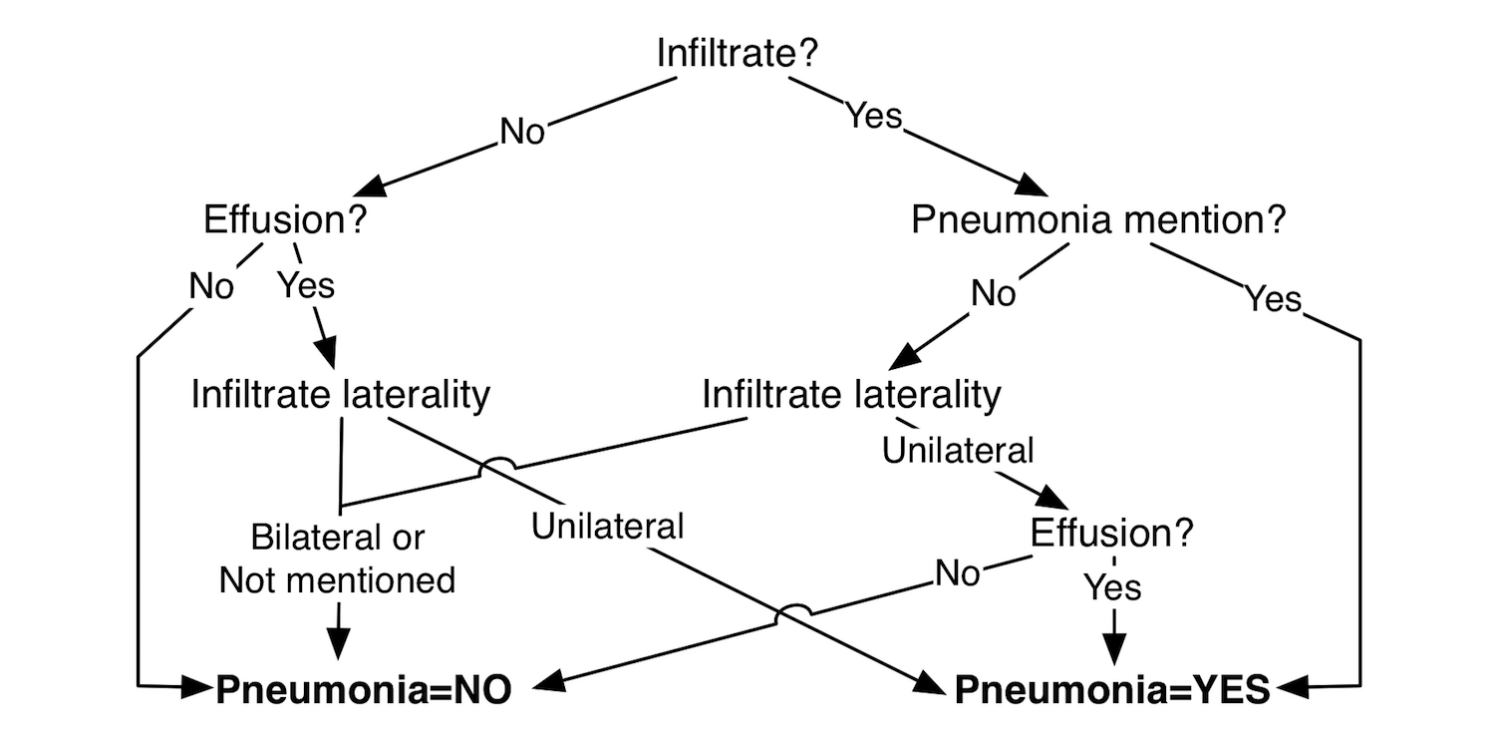
Pruned decision tree for pneumonia classification.

The decision tree algorithm (pruned C4.5 decision tree [33]) automatically created the decision tree and allowed for a classification *F*_1_-measure of .552 (Figure 4).

The rule learner (Repeated Incremental Pruning to Produce Error Reduction [RIPPER] [34]) automatically learned three rules that allowed for a classification *F*_1_-measure of .613:

- IF (Effusion=Present) AND (Symmetry=Unilateral) THEN Supports pneumonia=Yes
- IF (Infiltrate=Present) AND (Pneumonia mention=Present) THEN Supports pneumonia=Yes
- OTHERWISE Supports pneumonia=No

The Naïve Bayes algorithm implemented in Weka is based on John and Langley algorithm [35] and the Bayesian network implementation is based on several different algorithms such as Cooper K2 algorithm [36]. The Bayesian network allowed for the highest sensitivity (.739).

In Weka, the SVM implements John Platt's sequential minimal optimization (SMO) algorithm [32]. In our experiment, where the bag-of-words was not part of the features used here, it reached the highest positive predictive value (.811), but also had low sensitivity.

### Error Analysis

The most common errors our application made were false negatives, erroneously classifying reports as not supporting the diagnosis of bacterial pneumonia when they actually did support it. Among the 20 false negatives, most were cases of pneumonia that were not as clear, with only 48% of the expert annotators originally agreeing that they were positive cases. This average agreement was 86% for cases that were correctly classified. Most false negatives had no pleural effusion and some had infiltrates mentioned as “airspace disease,” which domain experts specifically decided to exclude as a clear indicator of bacterial pneumonia. Others had pleural effusions mentioned as “fluid” (without the mention of “pleural”), which were difficult to differentiate from other fluid locations in the thorax.

False positive errors (ie, erroneously classifying reports as supporting the diagnosis of bacterial pneumonia when they actually did not support it) were rarer, often caused by local context analysis errors (eg, “pleural effusion has completely resolved” not recognized as an absence of pleural effusion).

## Discussion

### Principal Findings and Comparison With Prior Work

The most accurate version of our NLP-based pneumonia information extraction application performed better than human domain experts, with significantly higher sensitivity (Fisher exact test, with *P*=.04.

We found variation in the language used in chest imaging reports both within and across the six children’s hospitals. This was due to inherent differences in imaging modalities, radiologists reporting, and hospital practice. Despite this variability in language, the most accurate version of our NLP-based diagnostic imaging reports classification application eventually reached a sensitivity of .71, positive predictive value of .86, and a specificity of .96. It was based on an SVM classifier with a refined set of features that included a filtered bag-of-words of 99 words, and the annotated concepts with their attributes. When tested in its first version, it only reached a sensitivity of .42.

Experiments to improve classification accuracy included refining the features and parameters used by the SVM classifier, and testing other algorithms. These algorithms included decision trees, rule learners, naïve Bayes, Bayesian networks, and SVMs. They allowed for sensitivity between .42 and .74, positive predictive value between .66 and .81, and specificity between .88 and .97. Even if the Bayesian network reached a slightly higher sensitivity than the most accurate version of our classifier (.739 vs .71), its positive predictive value was significantly lower (.78 vs .86), and the overall accuracy and *F*_1_-measure were therefore lower. These metrics are consistent with or significantly better than earlier studies such as the extraction of pneumonia information from chest radiology reports in a neonatal intensive care unit by Mendonça and colleagues [16], who reported .71 sensitivity but only .075 positive predictive value, or the extraction of pneumonia findings from chest radiology reports by Fiszman and colleagues [37], who reported .90 positive predictive value but only .34 sensitivity.

The performance reached by the most accurate version of our NLP-based reports classification application may seem low when considering the classification task it performed (ie, classifying diagnostic imaging reports as supporting the diagnosis of bacterial pneumonia or not), but this task was actually more difficult than it may appear. When comparing the three domain experts (ie, attending physicians) annotating these reports with the final reference standard, their average sensitivity was lower than the automatic classifier (Table 2). The positive predictive value and specificity were comparable. This comparison demonstrates the difficulty of the classification task, and the excellent performance of our application when compared with human experts.

### Limitations

Our evaluation had several limitations. First, although we had a small sample of annotated diagnostic imaging reports, this sample size allowed for CIs between .023 and .054 only (95% CI; Table 2). This pilot study only included imaging reports from 282 patients, but allowed for sufficiently precise assessment of the accuracy of our system to then apply it to a much larger population of more than 10,000 patients. Comparing our approach with domain experts would benefit from increased precision and could be based on an additional evaluation based on a new larger testing set. Next, the 5-fold cross-validation approach we used only yields meaningful results if the testing set and training set are drawn from the same population, which was our case (both were randomly drawn from our collection of diagnostic imaging reports). Cross-validation could also be misused if selecting features using the complete dataset, and using some data for both training and testing. We avoided both problems by selecting features manually (without examining the dataset, only the experts’ domain knowledge), and by ensuring that each report was used only exactly once for testing in our cross-validation approach. The BOW refinement process was purely manual and based on clinical domain knowledge, an approach that would not generalize easily to other applications. Finally, this pilot study was realized on a subset of clinical notes from a unique small population in 6 health care organizations, possibly making additional adaptations required to generalize to a larger population (eg, retraining the machine learning algorithms, refining the dictionaries used).

### Conclusions

We developed and used an NLP-based information extraction application to generate discrete and accurate data to identify pediatric patients with CAP. Our main objective was good positive predictive value and improved sensitivity when compared with human domain experts. The pneumonia information extraction application used methods and resources that were trained and evaluated with our reports collection, using a 5-fold cross-validation approach. It allowed for classifying pediatric diagnostic imaging reports with a higher accuracy than that by human domain experts (ie, higher sensitivity and similar positive predictive value and specificity) in this pilot study. After this study, it was used to extract information and classify a much larger collection of diagnostic imaging reports (more than 10,000) in the PHIS+ database, for subsequent community-acquired pneumonia research comparing the effectiveness of different treatment options.

## Appendix

Multimedia Appendix 1 Current Procedural Terminology Codes used to select relevant imaging studies.

Multimedia Appendix 2 Annotation guideline.

Multimedia Appendix 3 Refined bag-of-words.

Multimedia Appendix 4 Document level classification accuracy with different machine learning algorithms.

## Abbreviations

BOW: bag-of-words
CAP: community-acquired pneumonia
CER: comparative effectiveness research
CPT: Current Procedural Terminology
ICD-9-CM: International Classification of Diseases, 9th revision, Clinical Modification
NLP: Natural Language Processing
PHIS+: Pediatric Health Information System, augmented
PPV: positive predictive value
RBF: radial basis function
SVM: Support Vector Machine
UIMA: Unstructured Information Management Architecture
