# Supplementary material for: Enhancing Comparative Effectiveness Research With Automated Pediatric Pneumonia Detection in a Multi-Institutional Clinical Repository: A PHIS+ Pilot Study
Source: J Med Internet Res. 2017 May 15;19(5):e162. doi: 10.2196/jmir.6887 (PMC5447826; doi:10.2196/jmir.6887)
Supplement: Multimedia Appendix 1 [file jmir_v19i5e162_app1.pdf]

Appendix 1: Current Procedural Terminology Codes used to select relevant imaging studies.

**Concept description**	**CPT Code**
Computed tomography, thorax; without contrast material	71250
Radiologic examination, chest, special views (e.g., lateral decubitus, Bucky studies)	71035
Radiologic examination, chest, complete, minimum of 4 views; with fluoroscopy	71034
Radiologic examination, chest, complete, minimum of 4 views	71030
Radiologic examination, chest, 2 views, frontal and lateral; with fluoroscopy	71023
Radiologic examination, chest, 2 views, frontal and lateral; with oblique projections	71022
Radiologic examination, chest, 2 views, frontal and lateral; with apical lordotic procedure	71021
Computed tomography, thorax; with contrast material(s)	71260
Computed tomography, thorax; without contrast material, followed by contrast material(s) and further sections	71270
Computed tomography, thorax; without contrast material, followed by contrast material(s) and further sections	71270
Radiologic examination, chest; single view, frontal	71010
Radiologic examination, chest; stereo, frontal	71015
Radiologic examination, chest, 2 views, frontal and lateral	71020
Ultrasound, chest (includes mediastinum), real time with image documentation	76604
