# Supplementary material for: Enhancing Comparative Effectiveness Research With Automated Pediatric Pneumonia Detection in a Multi-Institutional Clinical Repository: A PHIS+ Pilot Study
Source: J Med Internet Res. 2017 May 15;19(5):e162. doi: 10.2196/jmir.6887 (PMC5447826; doi:10.2196/jmir.6887)
Supplement: Multimedia Appendix 2 [file jmir_v19i5e162_app2.pdf]

Annotation Guideline: PHIS+ Pneumonia Project

Introduction:

This document explains and provides guidelines for annotation of radiology reports that are relevant for the PHIS+ Pneumonia study. It also serves to maintain consistency across all annotators. The natural language processing tool will learn from the expert annotations and then automatically extract this information from clinical notes. Annotations will be done for each question (class) based on the research criteria for the pneumonia study.

A stratified random sample of 300 thoracic radiology reports (50 from each site) will be de-identified and provided for annotation. The annotation will be done using an annotation tool and the details will be explained on the scheduled annotation call. Each report will be independently annotated by two experts, and a third expert will adjudicate any disagreements.

Conventions applied to classes and slots below:

In the provided examples highlighted text provides examples of what to annotate. For some classes we have also provided examples of exclusions. Annotated information should include representations of the possessive form of proper names for example. For most annotated classes annotated information could include special characters such as -, /, (,), and includes the shortest span that completely captures all information representing each class and that can be separated by white space.

I. Pneumonia

1. Pneumonia (text span)

Annotate all mentions of pneumonia in the text (or synonyms - e.g., pneumonitis). Only annotate the noun, without any adjectives (except if required to define the concept; e.g., “lung infection” needs ‘lung’ to be precise enough).

Examples:

- Findings consistent with viral or reactive airways disease without focal pneumonia. **[Presence=No example]**

- Poorly defined opacities in left upper lobe likely represent developing pneumonia.

2. Presence (slot value)

For each pneumonia annotation, choose *Yes* if it is present (i.e., affirmed, not negated, current) in the reported images; *No* if it is absent (i.e., negated, excluded); *Speculative* if it is hypothetical (i.e., a possibility, to rule it out); or *Historical* if it is past and not current anymore, using the corresponding values of the Presence slot.

II. Pleural effusion

1. Effusion (text span)

Annotate all mentions of pleural effusion in the text (or synonyms - e.g., empyema; or terms that imply the existence of a pleural effusion if “pleural effusion” or a synonym is not mentioned – e.g., loculation, free fluid). Only annotate the noun, without any adjectives (except if required to define the concept; e.g., “lung infection” needs ‘lung’ to be precise enough). Only annotate ‘effusion’ in “pleural effusion.” Mentions of ‘effusion’ that are not pleural should not be annotated.

Examples:

- There are no pleural effusions. **[Presence=No example]**

2. Presence (slot value)

For each effusion annotation, choose *Yes* if it is present (i.e., affirmed, not negated, current) in the reported images; *No* if it is absent (i.e., negated, excluded); *Speculative* if it is hypothetical (i.e., a possibility, to rule it out); or *Historical* if it is past and not current anymore, using the corresponding values of the Presence slot.

3. Quantity (slot value)

   For each effusion annotation, indicate the quantity as *small*, *moderate-large*, or *no mention*, using the corresponding values of the Quantity slot.

III. Pulmonary infiltrate

1. Infiltrate (text span)

   Information annotated in this class includes all mentions of a pulmonary infiltrate (or synonyms like opacity, consolidation) in the text. Only annotate the noun, without any adjectives (except if required to define the concept; e.g., “lung infection” needs ‘lung’ to be precise enough; in “nodular infiltrate”, only ‘infiltrate’ should be annotated). Don’t include remote synonyms like “small airways disease”, “interstitial markings”, “peribronchial thickening”, or “atelectasis.” Abscesses should not be annotated.

   Examples:

   - There is a mild patchy opacity seen in the left lower lobe region
   - There is asymmetric increased opacity within the left lung base
   - Multifocal airspace disease, slightly worsened on the right.

2. Presence (slot value)

   For each effusion annotation, choose *Yes* if it is present (i.e., affirmed, not negated, current) in the reported images; *No* if it is absent (i.e., negated, excluded); *Speculative* if it is hypothetical (i.e., a possibility, to rule it out); or *Historical* if it is past and not current anymore, using the corresponding values of the Presence slot.

3. Laterality (slot value)

   For each infiltrate annotation, indicate whether it is *unilateral*, or *bilateral*, using the corresponding values of the Laterality slot.

IV. Overall pneumonia support

Annotate each document whether it supports the possible diagnosis of **bacterial** pneumonia (*true* value), or does NOT support the diagnosis of **bacterial** pneumonia (*false* value).

No text should be selected for this annotation. Simply add the annotation after the last character at the end of the report text.
