# Supplementary material for: Enhancing Comparative Effectiveness Research With Automated Pediatric Pneumonia Detection in a Multi-Institutional Clinical Repository: A PHIS+ Pilot Study
Source: J Med Internet Res. 2017 May 15;19(5):e162. doi: 10.2196/jmir.6887 (PMC5447826; doi:10.2196/jmir.6887)
Supplement: Multimedia Appendix 3 [file jmir_v19i5e162_app3.pdf]

Appendix 2: Refined bag-of-words

BOW1 (99 words)		BOW2 (37 words)		BOW3 (23 words)	
**Words associated with positive cases**	**Words associated with negative cases**	**Words associated with positive cases**	**Words associated with negative cases**	**Words associated with positive cases**	**Words associated with negative cases**
adjacent	abdominal	collection	mass	collection	mass
airspace	acute	patchy	axial	patchy	free
anechoic	administration	airspace	osseous	airspace	metastatic
associated	anterior	hemithorax	coronal	hemithorax	picc
atelectatic	aortic	significant	post	significant	procedure
bibasilar	arch	bibasilar	tissue	bibasilar	diffuse
cc	attenuation	parenchymal	placement	parenchymal	pericardial
collection	axial	consolidated	spleen	consolidated	uneventful
consolidated	axillary	opacification	free	opacification	radiation
debris	bilaterally	fever	abdominal	fever	
decrease	body	hazy	aortic	hazy	
decreased	bones	anechoic	interstitial	anechoic	
decubitus	bony	debris	tissues	debris	
evaluate	cardiomediastinal	level	metastatic	level	
fever	changes		vascular		
hazy	coronal		venous		
hemithorax	described		picc		
involving	diffuse		procedure		
largest	endotracheal		diffuse		
level	evaluation		pericardial		
limited	free		prominent		
lobes	glands		uneventful		
middle	helical		radiation		
opacification	hemidiaphragm				
parenchymal	interstitial				
patchy	intravenous				
possible	junction				
recent	line				
remains	mass				
represent	mediastinum				
residual	metastatic				
segment	osseous				
significant	pancreas				
simple	pericardial				
	picc				
	placement				
	portions				
	post				
	previously				
	procedure				
	prominent				
	radiation				
	region				
	sagittal				
	scattered				
	sections				
	single				

	spine<br>spleen<br>status<br>stomach<br>subclavian<br>subsegmental<br>superior<br>surgical<br>teaching<br>thorax<br>tissue<br>tissues<br>trainee<br>uneventful<br>vascular<br>vasculature<br>vein<br>venous				
