# Supplementary material for: Enhancing Comparative Effectiveness Research With Automated Pediatric Pneumonia Detection in a Multi-Institutional Clinical Repository: A PHIS+ Pilot Study
Source: J Med Internet Res. 2017 May 15;19(5):e162. doi: 10.2196/jmir.6887 (PMC5447826; doi:10.2196/jmir.6887)
Supplement: Multimedia Appendix 4 [file jmir_v19i5e162_app4.pdf]

Appendix 4: Document level classification accuracy with different machine learning algorithms

	Decision Tree	Rule Learner	Naïve Bayes	Bayesian Network	Support Vector Machine
**True positives**					
	29	38	48	51	30
**True negatives**					
	206	196	188	192	206
**False positives**					
	7	17	25	21	7
**False negatives**					
	40	31	21	18	39
**Sensitivity**					
	0.420	0.551	0.696	0.739	0.435
**Positive predictive value**					
	0.806	0.691	0.658	0.708	0.811
**F_1-measure***					
	0.552	0.613	0.676	0.723	0.566
**Specificity**					
	0.967	0.920	0.883	0.901	0.967
**Accuracy**					
	0.833	0.830	0.837	0.862	0.837
